# Climate change and cancer: an oncology nurse perspective in two Colombian regions

**DOI:** 10.3332/ecancer.2023.1620

**Published:** 2023-11-09

**Authors:** Natalia Martínez Arias, Ángel Alfonso Aguirre Durán, Mayra Yiseth Ramírez Lozano, Celia Díez de los Ríos de la Serna, Mar ía Fernanda Olarte-Sierra, Julia Challinor, Yuli Vanessa Girón Arbelaez, Magali Yolima Mera Díaz, Luz Damaris Rojas Rodríguez

**Affiliations:** 1 Nursing Program, Faculty of Health Sciences, Central Unit of Valle del Cauca, Carrera 27A, Tuluá 763021, Colombia; 2 Faculty of Health Sciences, Central Unit of Valle del Cauca, Carrera 27A, Tuluá 763021, Colombia; 3 School of Nursing, Faculty of Medicine and Health Sciences, Bellvitge Campus, University of Barcelona, Barcelona 08001, Spain; 4 Medical Anthropology and Global Health, Institute for Social and Cultural Anthropology, University of Vienna, Universitätsstraße 7, 1010 Vienna, Austria; 5 School of Nursing, University of California, San Francisco, CA 94102, USA; 6 Nurse Specialist in Adult Oncology, Hospital Education Program Leader, Fundación Valle del Lili Hospital, Cali 760002, Colombia; 7 Paediatric Oncology Unit, Somer Clinic, Rionegro, Antioquia 054047, Colombia; 8 Researcher and Director of the Healthcare and Society Group, Nursing Program, Faculty of Health Sciences, Central Unit of Valle del Cauca, Carrera 27A, Tuluá 763021, Colombia

**Keywords:** climate change, cancer, nursing care, communication, access

## Abstract

Given the lack of publications and public policies addressing the relationship between climate change and cancer care in Colombia, we present an exploration of the perspectives and communication practices of a group of nurses from Valle del Cauca and Antioquia. We provide a context based on the available literature on climate change and general health then provide an overview of cancer in the country. Next, we present how oncology nurses have incorporated information about strategies their patients can use to mitigate the effects of climate change on their health. We highlight the centrality of patient-centered communication using a framework from the US National Cancer Institute) and the fundamental role nurses have in patients' experiences throughout their treatment. We conclude with the need to investigate oncology nurse communication practices in other Colombian hospitals, with consideration of culture, cancer stigma, barriers to care and other factors that may influence successful climate change mitigation and to better understand how other Latin American oncology nurses are addressing this serious challenge.

## Introduction

In this article we present the perspectives and practices of nurses who are located in Valle del Cauca and Antioquia, Colombia. Three are teachers who prepare and manage nurses who provide direct care to patients with cancer (among other diseases) and two are clinical nurses in the (south)west of Colombia. First, based on the literature identified by the nurse educators, we present an overview of the climate change situation and health in general in Colombia followed by a brief description of the incidence of cancer in the country. This information forms the context for understanding the experiences of clinical nurses when managing cancer patients and their early attempts to address the impact of climate change on this vulnerable population.

Given the magnitude of the effects of climate change on health, we show that nurses working in Oncology have begun to incorporate information about cancer care in relation to climate change into their patient communication spaces. We used the model of patient-centred communication in cancer care settings developed by the National Cancer Institute (NCI) in the United States of America to emphasise the patient-centred communication that nurses describe.

### Cancer patient-centred communication

In order to understand how climate change [1] is affecting access to health services and the care of cancer patients in (south)western Colombia, we draw on the conceptual map developed by the NCI of the United States of America in the monograph ‘Patient-Centred Communication in Cancer Care: Promoting Healing and Reducing Suffering’ [2]. The main idea is to encourage and maintain cancer patient-centred communication in a way that is clear, simple, with room for complexity, and that addresses patients' needs, perspectives and beliefs, as well as conveying critical information regarding cancer diagnosis, treatment and prognosis. This framework comprises six overlapping and interacting core functions: fostering healing relationships, sharing information, responding to emotions, managing uncertainty, making decisions, and enabling patient self-management.

By using these core functions to present our findings in a structured way, we highlight the centrality of effective communication as a key element in achieving both good and desired outcomes in cancer treatment and management. This allows us to present the experience of a group of Colombian nurses in their frontline work with both cancer patients and nursing students on early interventions regarding the relationship between climate change and cancer.

## Method

This article is an exploration of climate change in Colombia and cancer from the perspective of five nurses (academic and clinical). The interest in investigating this topic is a result of the invitation to Colombian nurses to participate in this special issue and thus think about the possible implications that climate change had on their daily practice. In this sense, the findings presented here are framed as initial explorations that provide a basis for approaching the current state of research on climate change and health from the experience of health workers, meaning from their daily practice.

### Participants

Three of the nurses (co-authors) are teachers and are based in the city of Tuluá, Valle del Cauca. They have extensive experience in patient care and because of their experience, the hospital they belong to offers services to oncology patients. However, it should be noted that it is not a specialised cancer centre. Although their focus is on teaching, some of these nurses have supported the creation and maintenance of cancer patient support groups, especially for breast cancer patients. In this sense, even in academia and teaching, nurses are fully aware of the challenges and opportunities for cancer patients in this part of Colombia.

**Figure 1.**
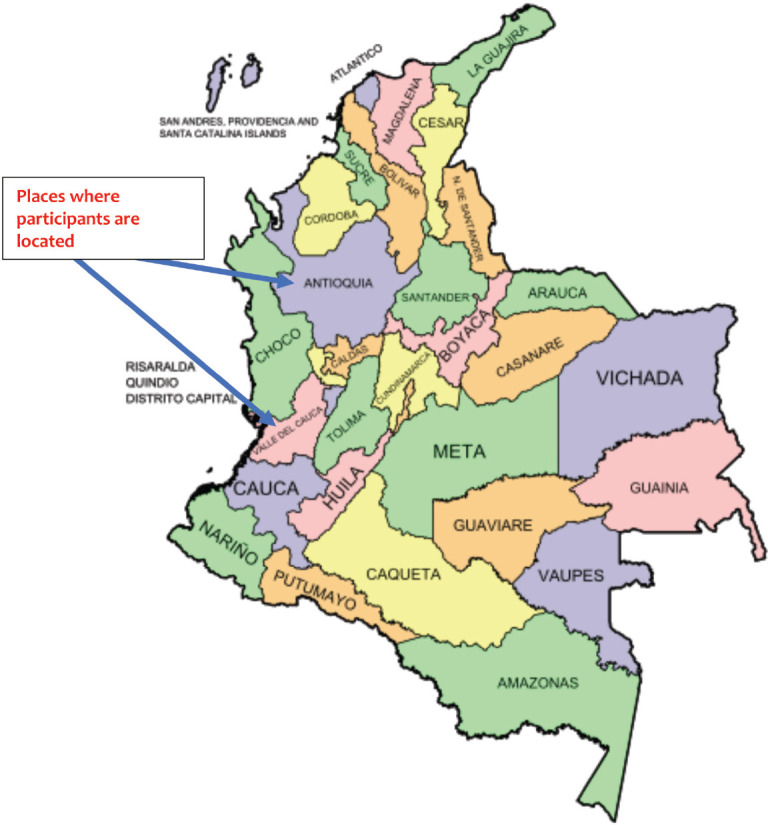
Map of capital district and departments of Colombia with Valle de Cauca and Antioquia identified [3].

On the other hand, two Colombian nurses (co-authors) are third level clinical oncology nurses and both specialise in oncology nursing. They shed light on the issues discussed here from their daily experience. One is located in a high-level clinic in the city of Cali (Valle del Cauca) and works with adult patients; the other is located in Rionegro (Antioquia) and focuses on paediatric oncology nursing (Figure 1). Both nurses are in leadership positions at the time of writing.

### Brief review of the literature

The three teaching nurses conducted a brief search of the available literature on the relationship between climate change in Colombia and cancer incidence and cancer patient care in academic search engines.

### Context interviews

To gain first-hand experience in these nurses, a medical anthropologist (co-author) with experience working with oncology nurses throughout Latin America conducted a group interview (lasting approximately one hour) with the teaching nurses and then with the two clinical nurses. The interview guide was created by a second medical anthropologist and bilingual senior oncology nurse from the USA. Who has extensive experience working in Latin America (co-author). The interview guide was designed to address the experiences and practices of nurses in their day-to-day dealings with climate change and cancer - the perspectives of their work and the experience of their patients. The interviews were conducted in Spanish, and with the consent of the participants, they were recorded. The interview was then transcribed by a professional transcriber.

Oral consent was obtained for the interview and the use of quotes to provide personal perspectives on climate change at the beginning of each interview. The medical anthropologists chose the appropriate quotes to insert in this document as well as the content of the article in consultation with the oncology nursing co-authors (see Appendix 1).

## Results

### Demographics, climate change and health in Colombia

**Figure 2.**
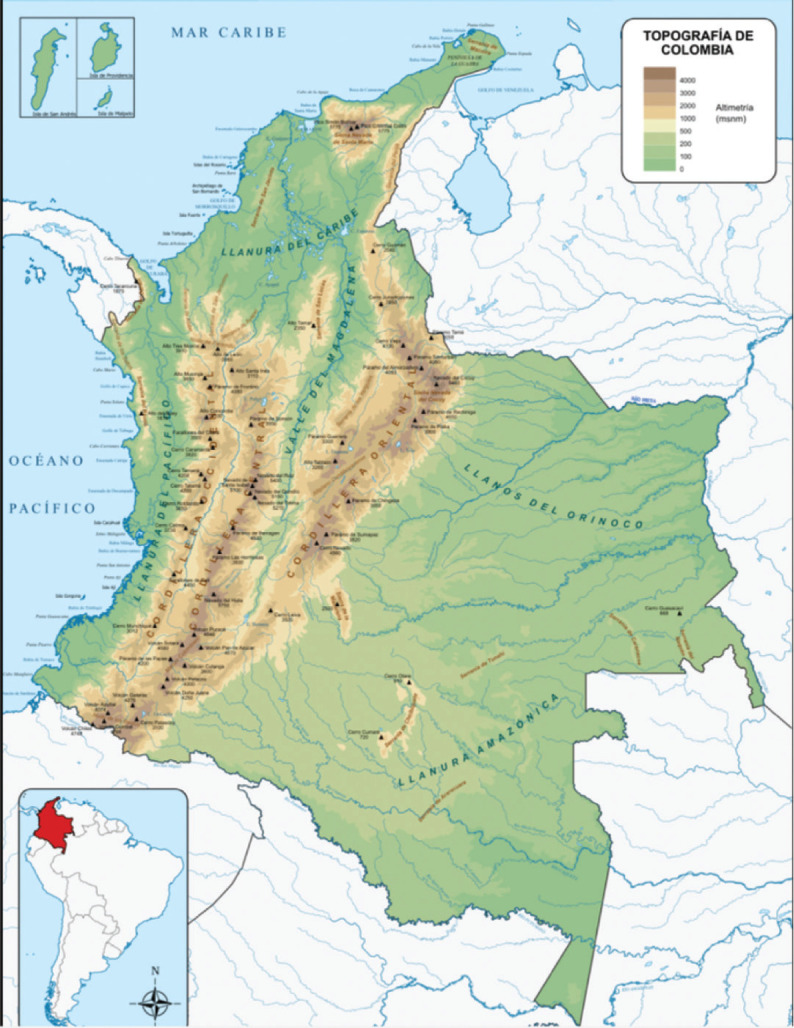
Topographical map of Colombia [4].

Colombia is a country in northwestern South America with coastlines along the Caribbean Sea and the Pacific Ocean (Figure 2).

The country has a population of 51 million people, [5] of whom 13 million are under 18 years of age [6]. According to its Constitution, the country is multi-ethnic and multicultural, with 84.2% of the population self-identifying as Mestizo, 10.5% as Afro-Colombian, and 3.4% as indigenous [7]. Around 40% of the population lives in poverty, which is exacerbated in areas of high rurality [8].

The Colombian territory does not have seasons, but periods of rain and drought depending on its location and proximity to the Andean Mountains, which, likewise, defined rainfall in non-established periods. The climate is defined by the thermal floors which, depending on the altitude, determine the humidity, temperature, vegetation and fauna that predominate in each one [9]. These conditions establish environmental, social, economic, labour and lifestyle characteristics, distances travelled and transport difficulties that can be determining factors in the process of health and illness of the Colombian population, particularly in terms of access to health services, as we will see below.

According to Feo *et al* [10], concerns about climate change and health were already identified in the Andean region more than 10 years ago.

It considers the direct and indirect implications of climate change for health, particularly in the Andean countries: disorders linked to the availability and quality of water and food, respiratory diseases, vector-borne infections, cancer and chronic degenerative diseases, cases associated with climatic disasters and extreme temperatures.

Similarly, Restrepo-Betancur *et al* [11] report how climate change in Colombia has had and continues to have adverse effects on the health of the population, such as vascular and respiratory tract diseases, including cancer, due to forest fires and poor air quality. Likewise, it has been reported that the increased sun exposure of some sectors of the population, whether for work or outdoor recreation, has led to a greater incidence of skin cancer [12, 13].

### Cancer in Colombia

The public health system is trying to address situations such as chronic non-communicable diseases (NCDs) that occupy an important place in the Region of the Americas; [14] within which, in Colombia, cancer is the leading cause of death [15]. It is estimated that in Colombia there are around 5,562 new cases of cancer annually, with a higher proportion in women [15]. In 2021, just under half of all new cancer diagnoses (41.1%) were in adults over 65 years of age and half of all new cases were in the subsidised category of the Colombian General Social Security System [15].

According to the estimated age-standardised incidence rates (globally) in 2020, it was shown that for Colombia the rate for all cancers, including both sexes and all ages, is 182.3/100,000 inhabitants [16]. The most common cancers in the country are breast, prostate, cervical, colon and stomach cancers, and these are prioritised in the 10-year plan for cancer control in Colombia [17]. Specifically, in the geographical setting where the nurses (local authors) of this article are located, Valle del Cauca, due to the increase in cancer rates, (e.g., 50% increase in breast cancer cases, and an increase of almost 80% in cervical cancer) the departmental government of Valle del Cauca adopted measures to reduce mortality figures by addressing early diagnosis and timely treatment of cancer [18, 19].

### Interviews

In examining the experiences and practices of nurses in (south-)western Colombia, we found that both the nurses themselves and the hospitals where they are employed are making localised attempts to communicate to their patients the importance of addressing climate change in the context of cancer. Despite the lack of a formal policy in Colombia [18] directly linking cancer to climate change, as emphasised by the teaching nurses, health workers are actively engaged in practical initiatives to address this issue in their daily routines.

It turns out that, this early care and attention work in the absence of public policy demonstrates the importance of cancer patient-centred communication. This is because, as noted above, it is necessary to maintain an open channel of communication that is sensitive to patients' needs, expectations, realities and perspectives. In this sense, we found that four of the six elements that make up the conceptual framework developed by the NCI and presented above are visible and shape nurses' practices regarding education about the effects of climate change on their patients' cancer care in Colombia.

In the following, we will address each of the four elements to make our findings evident.

### Fostering healing relationships

According to the conceptual framework presented, this core function refers to the need to establish and maintain relationships of trust, respect and understanding between patient and caregiver in order to achieve effective communication. As co-author Nurse Mera explains:

...little is explained to the parents [of paediatric oncology patients], step by step because it is a disease [cancer] that they do not expect, (...) for which they have no explanation as to why. And we as professionals, help them to organise their ideas and little by little, one will explain to them what they want to hear about the care they need to have and we speak about climate change (...) how the child can be exposed to the sun, what should be used, what clothes should be worn to protect the skin [due to the child's vulnerable state during cancer treatment] (Mera, July, 2023).

### Information exchange

In order to achieve effective communication, it is necessary to recognise the information needs that male and female patients have, in a way that integrates clinical aspects with explanatory models and patients' representations of the disease. In this regard, co-author Nurse Mera explains how care issues that include the effects of climate change are made explicit in cases of paediatric patients by their caregivers. In the words of Mera, ‘...parents ask [as a result of the treatment] what is going to happen to the skin, if they can withstand the sun, if they can go to the swimming pool (...) if the hair loss is going to cause injuries if the child is exposed to the sun. We explain to them that, although the child can go out, he should not be exposed to the midday sun’ (Mera, July, 2023).

In the case of older patients, the information is also adjusted to the activities that these people carry out, as co-author Nurse Girón points out:

We emphasize the use of sunscreen and sunglasses. We provide information sheets and follow-ups so that they keep the information in mind. We insist that when the older adults run their errands, go to the bank, or if they go to the store, they must always use sun protection (Girón, July, 2023).

### Managing uncertainty

Uncertainty is a recurring emotion in cancer treatment [2] and it is imperative that the patient is provided with support, information and cognitive tools to manage it. With respect to climate change, both co-authors Nurses Mera and Girón pointed out how, given the type of population their clinics serve, which includes rural and often low-income populations, inclement weather such as flooding due to rain, river overflows or landslides make access to health services difficult which delays treatments, putting the health of patients at risk. In this sense, within their communication practices and the management of uncertainty, is continuous contact with the patients and the reiteration of the need to continue with treatment even if an appointment has been missed. In addition, as Girón says, ‘we always try to ensure that our patients can attend, despite floods and landslides [increasingly frequent due to climate change]’ (Girón, July, 2023).

Similarly, co-author Nurse Damaris Rojas explains that part of her support and communication practices with the oncology population includes facilitating support groups and meetings for patients to exchange experiences and support each other on issues not limited solely to the effects of climate change: Rojas explains:

For example, we have supported the creation of self-help groups, especially for breast cancer patients. It is an emotional, economic and social support group. It is called Renacer [Rebirth] and everyone in that group supports and helps each other. We have 12 patients at the moment (Rojas, December, 2022).

### Allowing patient self-management

This element includes building relationships with the patient in which their interests are taken care of, where they are supported and accompanied in navigating the care system; as well as fostering patient autonomy by providing information, recommendations, instructions and access to resources. For example, co-author Nurse Mera explains that when giving recommendations, the socio-economic and working conditions of individuals are taken into account. She says: ‘ we know that a farmer who is exposed to sun all day, this person is not told not to expose himself, rather he is told how to prevent skin cancer, to hydrate, to use sunscreen, the clothes to wear’ (Mera, July, 2023). Similarly, Girón highlights that:

...we have many self-care campaigns where we inform the population of both the hospital as well as the general population about certain practices that can be followed to reduce the risk of cancer. For example, we give away sunscreen and show the correct way to apply it; but we also have activities to raise awareness about climate change and we teach about recycling through activities, programmes such as turning off the light when not in use and other similar activities that patients can do at home (...) (Girón, July, 2023).

On the other hand, the nurses indicated that one of the biggest barriers cancer patients face are delays in access that hinder timely diagnosis. The holistic care of these professionals and the nature of the communication in which they engage with their patients extends to providing support and legal assistance to enable patients to effectively access cancer services. Thus, structural issues of inequality and infrastructure or global issues such as the effects of climate change, won’t continue to affect mainly the most vulnerable populations. Nurse co-author Rojas expressed it like this:

[we have thought] How can we have an impact from the academy? The guideline we are thinking about is: let us empower the population with their rights and obligations. Here [in Valle del Cauca] we have the Central Unit of Valle and the idea was to ally with the Law School and the Doctor [Dean of the Nursing School],*why don't we join forces with the lawyers and tutor all the patients?,* because that's what needs to be done, let’s empower the patients and their families to claim their rights! (interview December, 2022)

## Discussion

In Colombia, the influence of mortality, morbidity and health effects on NCDs due to high precipitation, droughts, ‘atmospheric pollution, increased occurrence of food-borne diseases and acute diarrhoeal diseases, as well as diseases spread by rodents and vectors’ have been studied [19]. Thus, the role of climate change in the development of NCDs such as cancer, is increasingly being addressed both locally and globally, and it is known that environmental and climatic conditions in each region lead to population vulnerability depending on the geographical area in which they are located.

This is described by research on climatic and geographical conditions on factors in breast cancer in Iran, where they show that genetic, environmental and geographical factors such as UV exposure time lead to endocrine disruption and exaggerated vitamin D increase, i.e., a reduction in exposure time can minimise the risk of breast cancer [20]. On the other hand, high-income countries (e.g., Australia and the United Kingdom) are reducing both incidence and mortality rates due to early detection and access to better treatment [21].

However, these trends are not shared by low-and middle-income countries, such as Colombia, where there are large inequalities in access to health services, caused not only by the type of insurance, but also by certain social and demographic characteristics that affect access to health service provision [22]. In addition, as noted above, Colombia has made progress in understanding the relationship between climate change and the increase in cancers such as skin and respiratory tract cancer [13]. However, how health care workers are managing and coping with these realities with their cancer patients remains understudied. A literature review published by Hiatt and Beyeler [21], highlights the effects of climate change on some types of cancer, in relation to exposure to environmental and air pollution, ultraviolet rays, as well as failures in the supply of safe water and food, among other aspects.

### Communication

Using the conceptual framework previously presented to organise our findings, we were able to demonstrate the early actions that women are taking to mitigate the effects of climate change on cancer care and access to health services. We focus on patient-centred communication, given that it is one of the essential areas of nursing care, as it includes educational aspects, information, the provision of practical and cognitive tools, as well as looking after the interests of the patients [2].

Considering the nature of these practices, we based our findings and analysis on the conceptual framework of cancer patient-centred communication developed by the NCI. We show how the Colombian nurses’ communication processes presented here approaching the relationship between climate change and cancer care are characterised by four of the six elements that make up the conceptual framework presented, which allows us to point out their importance and potential effectiveness and efficiency. These four of the six main functions are as follows.

- *Fostering healing relationships*, where nurses know their patients and thus can build trusting relationships in which information is provided in a gradual and responsive manner to their information needs.
- *Information exchange* to provide information relevant to the patients’ situation, understanding their realities and assessing their needs.
- *Manage uncertainty* by providing tools that allow a better understanding of care and precautions regarding climate change in relation to the health status of patients. Also, supporting patients who for various reasons, including inclement weather, are unable to attend their chemotherapy and follow-up appointments. This support translates into incentives to continue attending despite having missed a session, as well as follow-up through telephone support.
- *Enabling patient self-management* through self-care campaigns, specific practices in hospitals that educate about strategies to contribute to the maintenance of the environment, and legal support to ensure that patients receive the treatment they need from health care institutions. In this way, the nurses look after the interests of their patients.

Graetz *et al* [23] found that there is a hierarchical and distant relationship in paediatric oncology between health professionals and families. In these contexts, the role of the nurse is particularly crucial, as they often have a closer socioeconomic status than physicians, especially in Latin America. This also highlights the importance of nurse-led support groups, as mentioned by one nurse in this study. By creating a trusting and supportive environment for cancer patients, nurses can broaden their discussion of cancer treatment alone and address additional topics such as climate change and the impact on cancer patients. Graetz *et al* [23] recommend research into multiple factors beyond medical hierarchies (i.e., political systems and religious traditions) and that is not based solely on definitions of patient-centred communication from high-resource countries. This suggestion recognises the key role of multiple factors which influence cancer treatment and care in resource-limited settings such as Colombia. In the case of Colombia, where there are great inequalities in access to health services, caused not only by the type of insurance, but also by certain social and demographic characteristics, which are marked by ‘....accessibility to the provision of health services, inefficiencies in their organisation and operation due to the deficit of health resources and the scarce supply in non-urban areas’ [22], or which favours not only the progression of the disease, complications and the failure to detect it early, but also the abandonment of treatment, the deficit in the quality of care and the failure to comply with public policies for health promotion and maintenance.

Understanding the health system, cancer incidence and access to care and successful treatment in Colombia, is therefore critical to making progress in addressing climate change and its known impact on cancer patients. The value of these practices, which we consider to be early, given that there is still no Colombian public policy of action that seeks to address the effects of climate change on cancer care, lies in the ability that nurses have developed, to know the needs, expectations and realities of their patients and in this way, provide information that is relevant and valuable to their patients.

## Conclusion

Concerns about the relationship between climate change and care and access to health services for cancer patients in Colombia are still rare. The country has no established health policies that address the effects of climate change such as high temperatures, increased ultraviolet rays, pollution, among others, on the health of the population, including cancer [13]. However, as shown in this article, this group of nurse co-authors have initiated, both spontaneously in some cases and supported by their clinics and hospitals in others, practices to inform and raise awareness among oncology patients about the risks that climate change can have on their health.

The central communication functions, although early and not yet detailed in a national plan, make evident the growing interest and undeniable need to address the relationship between climate change and cancer. However, further research on nursing communication practices is needed to gain a broader understanding of how this situation is being addressed in other cancer treatment settings in the country.

## Conflicts of interest

The authors state that this study did not pose any conflict of interest.

## Funding

This exploratory study did not receive funding of any kind and the authors did not receive financial compensation for participating.

## Appendix 1. Interview guide.

1. Could you tell me about the care of cancer patients in Valle de Cauca?
2. What is the difficult part of caring for a cancer patient?
3. What kind of work does your patient do or if he/she does not have a job, what are the opportunities?
4. Do the patients you see know about cancer and have they had a family member with cancer, what do they think cancer and treatment are?
5. What is the likelihood that the patient will complete all treatment on time?
6. How do patients find information about their cancer? (Are nurses teaching them? What is the nursing follow-up once the patient starts treatment?)
7. Do you have any radiotherapy facilities in your hospital or locality, is it covered by the government?
8. Do you have specialist surgeons for cancer surgeries?
9. Do you have rehabilitation services?
10. Do you have survivor monitoring?
11. What do you see that is related to climate change in the time you have been working in this hospital?
12. What are the biggest challenges in caring for cancer patients?
13. How do you treat children with cancer?
14. What are your greatest strengths in caring for cancer patients?
15. What would you change or improve if you could in your environment?
